# Narrative Podcasts to Foster Empathy and Reduce Stigma Among Third-Year Medical Students

**DOI:** 10.1007/s40596-023-01764-y

**Published:** 2023-03-14

**Authors:** Sara Powers, Wendy Craig, Michael Kohut, Anne Hallward

**Affiliations:** 1 Spring Harbor Hospital, Westbrook, ME USA; 2 Maine Medical Center, Portland, ME USA; 3 Maine Medical Center Research Institute, Scarborough, ME USA; 4 Tufts University School of Medicine, Boston, MA USA

Empathy and compassion are vital elements of the doctor-patient relationship. Higher levels of physician empathy positively impact patient satisfaction and adherence with recommendations, clinical outcomes, and rates of physician burnout [1–4]. Nonetheless, studies consistently reveal a decline in empathy during medical training, particularly during the clinical rotations of medical school [5].

Lower levels of empathy also correlate with higher levels of stigma about mental illnesses [6]. Mental health–related stigma may not improve over the course of medical training, even after participating in a psychiatry clerkship; this is worrisome, as medical students are already susceptible to stigma toward their patients [7–9]. Stigma has negative effects on self-esteem, income, employment, housing, interpersonal relationships, and healthcare, and increases suicide risk among those with mental illness [5]. These consequences argue for curricula addressing both mental health stigma and empathy in medical training.

Studies of anti-stigma interventions suggest that personal contact with individuals with mental illness, including stories of recovery, reduces mental health–related stigma among medical students [10]. Most third-year psychiatry clerkships, however, occur in inpatient settings, emphasizing acute decompensation without exposure to experiences of recovery. While podcasts are being used in psychiatry clerkships, narrative podcasts have not yet been evaluated as a form of contact to reduce stigma and foster empathy by introducing medical students to patients’ stories of illness and recovery [11]. We therefore designed narrative podcasts that included stories of recovery into the curriculum of the third-year medical student psychiatry clerkship with this in mind. The aim of this study was to explore students’ thinking about empathy and stigma after listening. We discuss quantitative and qualitative feedback from medical students and how this may guide future implementation of these podcast modules.

## Designing the Podcast Series

Narrative podcasts offer contact with stigmatized populations and invite the listener to hear personal stories without the guests needing to be physically present. This makes them easier to schedule, less expensive, and less burdensome for the storytellers. They are described as “narrative” because they are story-based, intimate, and vulnerable conversations broaching subjects that are difficult to talk about. They are not didactic in tone. Our hope was that as people with mental illnesses share very human stories of vulnerabilities and triumphs, listeners may consider them more “like me,” a process that itself fosters empathy [12].

We created five podcasts for each of the 5 weeks of the third-year psychiatry clerkship based on radio interviews by the Maine-based non-profit, Safe Space Radio. This is a nationally broadcast, public radio show whose mission is to reduce stigma, shame, and isolation and foster compassion and public health. These hour-long podcasts addressed five topics: Living with Major Mental Illness; Addiction; Suicide; Living with Anxiety; and Shame and Trauma in the Medical Encounter. Each episode contains both patient and family testimony along with expert reflection about the subject.

A 2019 review of podcast use in psychiatric education identified possible areas of controversy including discussion of non-evidence-based treatments, controversial opinions, and focus on treatment failure [11]. We avoided these pitfalls by creating the podcasts from carefully selected stories from a 300-episode archive hosted by a board-certified psychiatrist (AH).

## Implementing the Podcast Series

As a requirement of their clerkship, from March 2020 to March 2021, all medical students rotating through their third-year psychiatry clerkship at Maine Medical Center were expected to listen to one podcast a week. The podcasts are supplemented by an online module for each episode that includes learning objectives, discussion questions, additional resources, and post-test questions. There was a weekly group meeting with the clerkship director, who facilitated discussion of the podcasts by inviting students to share their thoughts and reflections. The podcasts are available for public use and can be accessed at www.safespaceradio.com/education.

## Measuring Impact

The first and last authors developed a questionnaire for this study with ten Likert-scale questions to explore whether listening helped them identify with people who struggle in this way, whether it motivated them to learn more about the subject, and whether it increased their confidence to address these subjects with their patients. It also included three free-text questions designed to make explicit whatever previous stereotypes or assumptions about people with mental illness they may have been carrying. We also asked them to identify key clinical takeaways and how they plan to make changes in their clinical work.

Learners were asked to complete this weekly questionnaire, which was voluntary and anonymous. Most students had the opportunity to complete a survey for each of the five podcast modules; one group participated for only 3 weeks due to the COVID-19 pandemic. This study was reviewed as exempt by the MaineHealth Institutional Review Board.

In this mixed-methods approach, we applied both descriptive quantitative analysis and inductive thematic analysis to our survey results. Inductive thematic analysis followed Braun and Clarke’s iterative, 6-step process to identify, evaluate, and relate themes in student free-text responses [13]. All four authors independently coded responses, then met to review codes and generate themes that were evident across surveys and questions.

## Outcomes

During the year, 44 students participated in the rotation (212 person-weeks). Eighty-two surveys were completed out of 212 opportunities (38.7% response rate) (Table 1). Thirty-one students (37.8% of total) responded to the first podcast in the series, Major Mental Illness. The clerkship director noted that the first two podcasts tended to generate lively discussion and even some self-disclosure; by the third week, however, there was less engagement with the podcasts in their weekly meetings. Approximately two thirds of the way through the year, we noticed this pattern and deliberately reversed the order of the podcasts to improve feedback about later topics.

**Table 1:** Participant responses to survey questions after listening to weekly narrative podcast episodes

Questionnaire items	*n*	Frequency, *n* (%)	
		Strongly disagree/disagree	Strongly agree/agree
Was beneficial for my medical education	81	3 (3.7)	78 (96.3)
Was interesting and engaging	81	3 (3.7)	78 (96.3)
Helped me identify with people who have this particular struggle	80	3 (3.8)	77 (96.2)
Will help me provide better care for patients	80	4 (5.0)	76 (95.0)
Made me feel more prepared to work with individuals sharing similar experiences	81	5 (6.1)	76 (93.9)
Increased my knowledge about these topics	81	6 (7.4)	75 (92.6)
Motivated me to learn more	81	6 (7.4)	75 (92.6)
Recommend module to other medical students	82	6 (7.7)	72 (92.3)
Increased my confidence to bring up this subject with patients	81	11 (13.6)	70 (86.4)
Increased my interest in working with this population	79	12 (15.2)	67 (84.8)

Overall, students were highly positive about the educational value of the podcast series. In particular, 96.2% of responses indicated agreement or strong agreement that, after listening to the podcast, they felt better able to identify with the person struggling, a measure of both empathy and stigma reduction. The majority also reported feeling more prepared to work with these patients (93.9%), increased knowledge about the topic (92.6%), increased motivation to learn more about the subject (92.6%), and greater confidence in bringing up these sensitive topics (86.4%).

We identified three overlapping themes while analyzing students’ free-text responses. Table 2 summarizes these themes and sub-themes, in addition to providing examples of prior assumptions and key clinical takeaways.

**Table 2:** Themes and examples from the students’ free-text questions

Themes	Sub-themes	Examples of previous assumptions that were challenged^1^	Reported takeaways and intended changes to practice^2^
Empathy	Us vs. them, “othering”	People with mental illness are low functioning, irrational, and possibly dangerous, not like “us”	To feel hopeful for many of the patients I have already seen this week
	Impact on the family	As a provider, my primary focus is the patient	Really listen to understand the patient and family’s experience
	Patients’ struggles with stigma	Stigma is abstract and does not have a real impact on life	Approach my patients with a different attitude, appreciating the societal challenges/systemic issues of having a mental illness (separate from the illness itself) like stigma, lack of affordable care, and housing
	Challenge of accessing care	Treatment is easily accessible and available	
Stigma Reduction	Moral judgment	Addiction is a choice	Hear patients out first rather than jumping to conclusions
	Taboo subjects	Suicidal thinking cannot be talked about, it might make things worse	Ask about difficult feelings
	Hope for prognosis	Poor outcome is inevitable	See mental illnesses as treatable medical conditions
Medical Humility	Harms of treatment (restraints, diagnoses, warehousing, language like “commit”)	The medical system is always benign	Be mindful of the language of diagnosis and the trauma of hospitalization
	Limits of treatment (chronic illness, the power of addiction)	Once the patient is in treatment, the biggest challenges are over	Appreciate that addiction is a lifelong challenge (despite treatment)
	Medical knowledge vs. patient knowledge	My medical knowledge is more valuable than the patient’s experience	Recognize and honor that the patient is the authority on their own experience

^1^These data were in response to the first open-ended question, “What was one assumption that you had about these individuals (or their families) that was challenged?”

^2^These data were in response to the following questions, “What was the most important takeaway for you?” and “What is one thing you would do differently with your patients in the future?”

Our first theme—empathy—captures how learners came to recognize a common humanity with patients. Students recognized that, prior to listening, they erroneously saw individuals with mental illness as fundamentally different from themselves. In empathizing, they appreciated the significant impact of stigma on their patients’ lives and the structural and societal challenges to accessing mental health care. Respondents resolved to bring a more understanding attitude to interactions with patients and families.

Our second theme focused on stigma reduction. Learners reported the podcasts encouraged them to see mental illness as a medical condition as opposed to a choice or moral failing. They revised their assumptions that people with mental illness are hopeless, adopting more hope for their prognoses. They recognized how not talking about these subjects reinforces the stigma surrounding them, resolving to initiate these conversations with their patients.

While these two themes—empathy and stigma reduction—reflected the focus of our study, a third—medical humility—emerged inductively through our discussion of the responses. Students introduced the theme of medical humility by reporting awareness of the capacity of medical treatment to harm (e.g., warehousing) and the ways that common medical language can be shaming to patients. They also recognized the limits of treatment and that many mental illnesses are lifelong conditions despite good treatment. Finally, recognizing the limits of medical knowledge, they reported plans to listen to and include the patient’s experiential knowledge in shared decision-making.

## Conclusion

We designed this narrative podcast series as a tool to augment the psychiatry clinical curriculum with the ultimate goal of fostering empathy and reducing mental health–related stigma in future medical providers. By providing a form of contact with families and individuals struggling with mental illness and their stories of recovery, the podcasts offer an important counterbalance to traditional psychiatric education in the inpatient setting. Student feedback provided strong support for the podcast series. Notably, they reported that listening to podcasts helped them identify with people struggling with mental illnesses, which can be seen as an important outcome of both perspective taking and developing empathy for members of stigmatized groups. The podcast series continues to be used at our institution and is being implemented at other sites around the country.

The podcast medium has several advantages as a medical student teaching tool. Audio-only stories invite the listener to imagine the scene and the characters, which facilitates the imaginative stretch of putting oneself in their shoes, an integral component of perspective taking. Perspective taking is a component of cognitive empathy and has been shown to predict empathy in future physicians [11, 14]. Furthermore, voice-only communication may lead to higher rates of empathic accuracy than screen-based modalities [15]. Lastly, the prerecorded audio format allows for individuals with mental illnesses to share vulnerable stories without the strain and visual exposure from repeated in-person visits for cycles of medical students.

Initially, we were focused on the outcomes of empathy and reduced stigma, not on medical humility. An emerging literature supports the benefits of physician humility for patient outcomes, patient satisfaction, and trust [16]. Humility includes having an accurate view of one’s own strengths and limitations, an openness to learning, and an egalitarian approach to others [17]. Together, empathy, non-stigmatizing attitudes, and humility each reflect a quality of openness, receptivity, and respect for the vulnerable experiences of others as fellow humans.

Despite 84.8% of students reporting that the podcasts increased their interest in working with this population, 15% of students disagreed. It could be that students interpreted the question to be asking whether they were more likely to want to go into psychiatry, yet many students already have strong preferences regarding specialty choice. Another challenge was that survey feedback from medical students declined as their clerkship progressed. This may have been due to competition from mandatory activities, such as preparation for the end of clerkship exam. Future use of these podcasts might benefit from making feedback a requirement.

Our study had several limitations. Our study design did not use a formal empathy or stigma scale to compare pre- and post-listening data. Rather than measuring students’ personal levels of empathy, we were more interested in their feedback about the educational value of the podcast, including how it had made an impact on their attitudes and assumptions. Despite the overwhelmingly positive responses that we collected, we did have a low response rate to the voluntary survey. While this does not necessarily imply they did not listen to the podcast, it suggests that they may not have prioritized filling out the survey among other tasks. As responses were anonymous, we were also unable to identify how many of these students listened to more than one podcast. Finally, as this is a single-site study, generalizability is unknown.

This educational, narrative podcast series included in the psychiatry core clerkship was well received by students, with the potential to reduce stigmatizing assumptions and to foster perspective taking as an element of empathy. It is easy to implement, as this resource is freely available online and can be integrated into the psychiatry clerkship with weekly discussion groups. While future work is needed, the results are promising, as they meet our goal of creating a scalable assignment that promotes empathy and is of interest to learners.
